# Five-Year Outcomes in Bariatric Surgery Patients

**DOI:** 10.3390/medicina56120669

**Published:** 2020-12-03

**Authors:** Olga Nedeljkovic-Arsenovic, Marko Banovic, Dejan Radenkovic, Nemanja Rancic, Snezana Polovina, Dragan Micic, Ivana Nedeljkovic

**Affiliations:** 1 Department of Cardiology, Clinical Center of Serbia, 11000 Belgrade, Serbia; ivannanedeljkovic@yahoo.com; 2 Medical Faculty, University of Belgrade, 11000 Belgrade, Serbia; markobanovic71@gmail.com (M.B.); dejanr09@yahoo.com (D.R.); dragan.micic@med.bg.ac.rs (D.M.); 3 Centre for Clinical Pharmacology, Military Medical Academy, 11000 Belgrade, Serbia; 4 Faculty of Medicine of the Military Medical Academy, University of Defense, 11000 Belgrade, Serbia; 5 Department of Endocrinology, Clinical Center of Serbia, 11000 Belgrade, Serbia; snezanapolovina@gmail.com; 6 Faculty of Pharmacy, University of Novi Sad, 21000 Novi Sad, Serbia

**Keywords:** obesity, bariatric surgery, cardiovascular risk, long-term follow-up

## Abstract

*Background and objectives:* Obesity presents as a multifactorial, pandemic disease that arises as a consequence of unequal energy intake and energy consumption. Obesity adversely affects the quality of life, leading not only to disability, but also to various other disorders. Bariatric surgery is the most effective method for achieving significant and sustained weight loss in individuals with extreme obesity. The aim of this study was to examine how well surgically induced weight loss is maintained after five years of follow-up and its effects on cardiovascular risk factors and outcome. *Materials and Methods:* This is a retrospective cross-sectional study of 66 patients with morbid obesity, with body mass index (BMI) ≥ 40 kg/m^2^ or BMI ≥ 35 kg/m^2^ and obesity-related health conditions, aged 20 to 61 years, mostly women (77.3%) who underwent laparoscopic Roux-en-Y gastric bypass surgery. *Results:* Average follow-up was 6.42 years (95% CI 6.30–6.54 years) after surgery, with survival rate of 97% in operated individuals. There was a statistically significant reduction of weight and body mass index 6 months and 5 years after surgery in comparison to the initial values (*p* < 0.001). Of 62 patients who presented weight loss at the end of the follow-up period, 38 were able to maintain the amount of weight loss that was attained 6 months after surgery, while 24 patients regained weight compared to their postoperative weight at 6 months. Two patients reported no weight loss after treatment. Significant weight reduction was associated with better control of diabetes and increased self-reported physical activity at 6 months and 5 years after surgery, as well as with a reduction of the use of anti-diabetic and anti-hypertensive medications. *Conclusions:* Our research demonstrates a positive long-term impact of bariatric surgery on patients’ health conditions, significant and sustained weight loss, and decrease in BMI, which were associated with a reduction of co-morbidities and risk factors for cardiovascular diseases.

## 1. Introduction

Obesity is a common metabolic disorder, and its prevalence is increasing. It presents as a major public health problem in the 21st century [1]. Obesity is considered to be a multifactorial, pandemic disease that arises as a consequence of an unequal balance between energy intake and energy consumption. Besides genetic predisposition, environmental and behavioral habits have been implicated as important causes of the obesity epidemic [2].

Obesity adversely affects the quality of life, leading not only to disability but also to various other disorders. Being overweight is a significant risk factor for mortality, high blood pressure, diabetes, smoking, and physical inactivity and is responsible for 5% of all deaths worldwide [3]. Adult obesity (body mass index (BMI) > 30 kg/m^2^) is associated with a shortening of life expectancy by seven years according to the results of the Framingham study [4], whereas morbid obesity (BMI > 40 kg/m^2^) is strongly associated with numerous co-morbidities and a shortening of life expectancy by 12 years. The same study suggests that losing weight by 10% would reduce the risk of cardiovascular disease by about 20% [4].

Bariatric surgery is the most effective method for achieving significant and sustained weight loss in individuals with extreme obesity [5]. Roux-en-Y gastric bypass (RYGB) presents the most common surgical operation in such patients. A restrictive effect on oral intake is achieved by splitting the stomach surgically into two parts, the proximal smaller part (the gastric pouch) and the other larger part that is permanently closed [6]. Malabsorption is accomplished by creating a long Roux-and-Y bypass that connects the gastric pouch with the proximal jejunum, usually at a distance 40–50 cm from Treitz’s ligament [6]. Bariatric surgery may provide benefit in the long term not only by reducing or eliminating obesity, but also by improving other quality-of-life metrics and the metabolic status, including better regulation of diabetes, blood pressure, and serum lipids [7,8].

Generally speaking, bariatric surgery is considered to be a safe intervention with low mortality and morbidity, whose rates are similar to those of interventions such as laparoscopic gallbladder surgery [9,10]. The estimated mortality risk for gastric bypass surgery is about 0.4%, whereas the risk for major postoperative complications is 3.4–5.1% [10,11]. Although extensive data demonstrate the safety of bariatric surgery, there are fewer data about the long-term impact of this surgery on cardiovascular mortality and alteration of other cardiac risk factors.

Thus, the aim of this study was to examine how well surgically induced weight loss is maintained after five years of follow-up and how the maintenance of weight loss after surgery affects cardiovascular risk factors and outcomes.

## 2. Materials and Methods

This is a retrospective cross-sectional study of 96 patients who were scheduled to undergo bariatric surgery. Of them, 66 patients fulfilled the inclusion and exclusion criteria and were selected for follow-up after surgery [12,13].

Local Ethical Committee approval was obtained. Sixty-six patients with morbid obesity with a BMI ≥ 40 kg/m^2^ or a BMI ≥ 35 kg/m^2^ and obesity-related health conditions were included in the study. The patients’ age ranged from 20 to 61 years, and the patients were mostly women (77.3%) [12,13]. Excluded patients were those who at the time of surgery had unregulated hypertension, coronary artery disease, significant valvular defects (especially aortic stenosis and mitral regurgitation), atrial fibrillation, severe obstructive pulmonary disease, a history of syncope, or who were non-surgical patients. All patients were examined at the Clinical Center of Serbia and all signed informed consent. We compared their weight, BMI, and co-morbidities prior to, six months, and five years after RYGB, as well as their global health status. The parameter used to determine changes in weight after bariatric surgery was percent excess weight loss (%EWL) using a BMI of 25 kg/m^2^ as “ideal”. It was calculated by the following formula: ([BMI at surgery − BMI at time of follow-up]/[BMI at surgery − 25 kg/m^2^]) * 100. All patients had their weight measured on an electronic Tanita scale type BC-401 (Tanita, Amsterdam, The Netherlands), and their height was assessed with an altimeter. All patients were provided recommendations to engage in physical activity after surgery by having a minimum one-hour daily walk. Routine follow-up after bariatric surgery consisted of office visits twice a year, at which time the patients were assessed for blood pressure, electrocardiogram (ECG), and weight; basic laboratory blood tests were performed to determine fasting glycemia, lipide profile, and the levels of urea, creatinin, transaminases, electrolytes. Type 2 diabetes mellitus (T2DM) was diagnosed with two results of fasting glucose ≥ 7.0 mmol/L. We considered as index of diabetes resolution normal fasting blood glucose values of 4.0–6.1 mmol/L. According to the current recommendations, patients after bariatric surgery take supplements to ensure proper nutrition following their procedure including a complete multivitamin supplement, Vitamin B12, Calcium with Vitamin D, Iron and Vitamin C, Vitamin D. These vitamins help them to meet their nutritional needs. All supplements were prescribed on an individual basis in concordance with the clinical status of the patients.

Statistical analysis was done using the statistical software package PASW Statistics 18^®^ (SPSS (Hong Kong) Ltd., Hong Kong). Categorical variables were presented as frequency of certain categories. The Chi-square test and McNemar test were used for analyzing the significance of the differences of categorical variables. Continuous variables are presented as median with interquartile range 25th–75th percentile or as mean with standard deviation according to data distribution and were compared using the nonparametric Wilcoxon Signed Ranks Test and Friedman test. Distribution normality was tested using the Kolmogorov–Smirnov test (the number of subjects was > 50). Overall survival estimates were calculated using the Kaplan–Meier method, and overall survival rates were presented as a mean (95% confidence interval (CI)). The patients who remained alive were censored at the cut-off date. All analyses were estimated at *p* < 0.05 level of statistical significance.

The principles of ICH Good Clinical Practice were strictly followed, and approval from the Ethics Committee of the Clinical Center Serbia was obtained for the study protocol under the number 318/6 on 18 May 2017.

## 3. Results

Patients who underwent RYGB surgery were followed for at least five years, with some of them receiving follow-up for a longer duration. The mean duration of follow-up was 5.67 ± 0.63 years.

**Figure 1:**
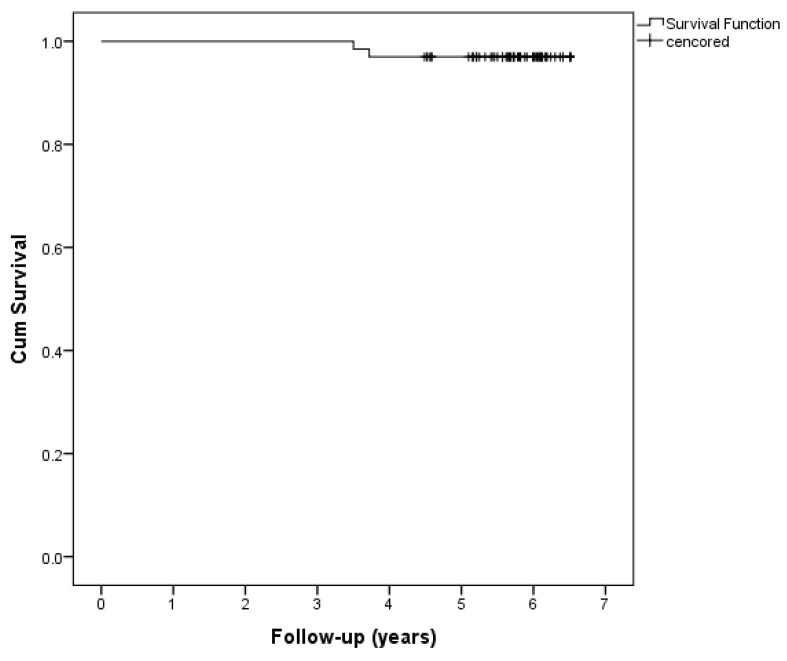
Kaplan–Meier analysis; survival curves of patients with extreme obesity after bariatric surgery (censored—alive at the end of the follow-up period).

Figure 1 presents a Kaplan–Meier survival curve demonstrating an average follow-up and survival of 6.42 years (95% CI 6.30–6.54 years) after surgery. At the time of study completion, all living patients were contacted and examined. During the study period, only two patients died, such that a five-year survival rate of 97% was reported in surgically treated patients. Both deaths occurred in year 3 after surgery, one due to carotid dissection, and the other following myocardial infarction.

**Figure 2:**
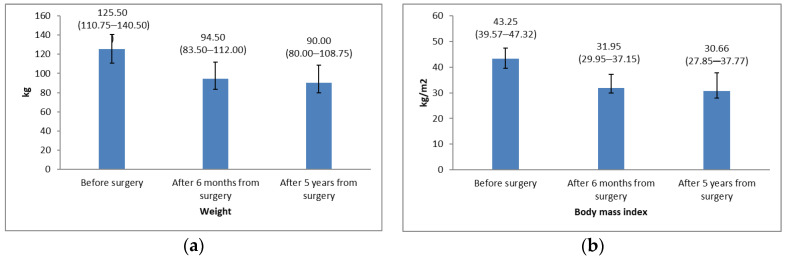
(**a**) Average values of weight before surgery, at 6 months, and at 5 years after bariatric surgery (Friedman test; *p* < 0.001); (**b**) Average values of body mass index (BMI) before surgery, at 6 months, and at 5 years after bariatric surgery (Friedman test; *p* < 0.001). In both figures, values are presented as median with interquartile range 25th–75th percentile.

Weight and BMI before and after surgery are presented in Figure 2. There were statistically significant reductions of weight and BMI at both 6 months and 5 years after surgery in comparison with the initial values (Wilcoxon Signed Ranks Test; weight: before vs. 6 months after surgery: *p* < 0.001, before vs. 5 years after surgery *p* < 0.001; BMI: before vs. 6 months after surgery: *p* < 0.001, before vs. 5 years after surgery *p* < 0.001). In addition to a statistically significant average body weight reduction after five years from surgical treatment, weight decreased an additional 4.5 kg between the 6 month and the 5 years’ time points. However, the change in average weight and BMI from six months to 5 years after surgery was not statistically significant (Wilcoxon Signed Ranks Test; *p* = 0.095, *p* = 0.086, respectively).

In comparing the body weight of the 64 followed patients 5 years after surgery and before surgery, 62 patients (93.9%) experienced weight loss (31.50 (20.00–44.25) kg), while 2 patients presented weight gain (4 and 7 kg). BMI was also lower at 6 months after treatment and further decreased at the 5-year assessment (Figure 2).

In assessing the degree of weight loss at the 5 years’ time point compared to the level of weight loss 6 months after surgery, of the 64 patients who were followed, 38 patients (57.6%) presented additional weight loss (12.00 (6.25–17.00) kg), while 24 patients (39.4%) had weight gain (9.00 (3.50–15.50) kg). 

Table 1 shows that of the 62 patients who experienced weight loss at the end of the 6-month follow-up period, 38 patients were able to maintain weight loss 5 years after surgery, while 24 patients regained weight compared to their weight 6 months after surgery. However, these patients’ weight did not return to their baseline values (McNemar test; *p* < 0.001). Of the initial cohort of 66 patients, only 2 patients presented no weight loss after treatment throughout the follow-up period.

**Figure 3:**
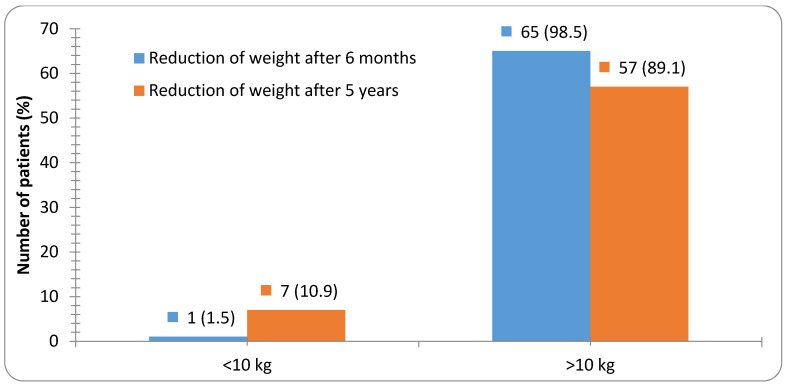
Number of patients with weight reduction 6 months and 5 years after bariatric surgery in relation to their weight prior to surgery.

Most patients (89.1%) presented > 10 kg weight loss at the 5 years’ time point in relation to their baseline weight prior to surgery, while only 10.9% of patients showed weight reduction of less than 10 kg in the same period (Figure 3).

Results were similar using %EWL as a recommended parameter of weight loss and bariatric surgery success. At 6 months after surgery, the %EWL was 55.38 (39.54–70.77), while at 5 years after surgery, the %EWL was 64.74 (33.10–83.15).

Risk factors and lifestyle changes before surgery and 5 years after surgery are shown in Table 2. The results indicate a decrease of T2DM prevalence as well as a reduction in the number of patients using antidiabetic drugs at 6 months and 5 years after bariatric surgery. However, after 5 years we are still treating insulin resistance in one patient. A statistically significant increase in physical activity was seen, especially at the 5 years’ time point. There was no change in the incidence of hypertension or use of antihypertensive drugs. Also, the use of statins for treating hypercholesterolemia remained unchanged after five years (Table 2). A concerning finding was the higher rate of smokers at the 5 years’ time point. The Chi-square test showed that there was no significant difference in the use of antihypertensive therapy between baseline, 6 months, and 5 years after surgery (*p* = 0.621). As for complications after surgery, none was reported.

## 4. Discussion

Our study has demonstrated that there is a favorable impact of bariatric surgery on certain cardiovascular outcomes and cardiovascular risk factors in patients with obesity. This study is unique in our country for its long-term follow-up, examining the effects of bariatric surgery on cardiopulmonary capacity and cardiovascular risk factors.

In terms of survival after surgery, we reported a high rate of surviving patients, with 97% of operated patients being alive at least 5 years after surgery [12]. There were only 2 deaths in 66 people over this period of time.

In a large Swedish prospective study of persons with obesity (SOS), during a follow-up period of 15 years, the overall mortality rate was 30.7% lower in the bariatric group compared with control subjects who did not undergo surgery. The most common causes of deaths reported were myocardial infarction and cancer [14,15]. In another large retrospective cohort study with median follow-up of 7.1 years, mortality from any cause was decreased by 40% in the RYGB surgery group compared with the control group [16].

A study by Flum and Dellinger examined short- and long-term outcome (up to 15 years) after bariatric surgery, showing a significant reduction in deaths from 16.3% to 11.8% in surgically treated patients compared to study controls who were not surgically treated [17]. In a meta-analysis of studies with an average of 14 years of follow-up after surgery, the incidence of cardiovascular events and cancer was found to be approximately half in the bariatric surgery group compared with the nonsurgical cohort [18]. According to a study by Christou et al., the mortality rate of patients who had bariatric surgery was only 0.68%, compared with 6.17% of the control, non-operated group [19].

A recent meta-analysis has shown that weight loss in patients with obesity with co-morbidities is associated with improvement in multiple domains of health, as well as with increased survival [20]. Additionally, the results of prospective studies of patients with obesity with co-morbidities demonstrated that weight reduction is associated with decreased total and cardiovascular mortality, primarily by lowering the prevalence of diabetes mellitus [21].

The lack of long-term follow-up has been reported as a limitation of such studies. Hence, we followed patients in our cohort for a minimum of 5 years.

Weight loss is considered one of the main parameters in evaluating the success of bariatric surgery. In our study, the mean preoperative BMI in men was 43 kg/m^2^ (median: 42.90 kg/m^2^), while in women it was 43.8 kg/m^2^ (median: 43.60 kg/m^2^). Following surgical treatment, at 6 months the BMI in men and women was reduced to 33.4 kg/m^2^ and 33.8 kg/m^2^ (median: 34.40 and 31.90 kg/m^2^), respectively [13]. Smith et al. [22] reported a mean preoperative BMI of 46.7 kg/m^2^ in their study, with BMI at 6 months postoperatively after laparoscopic RYGB of 33.1 kg/m^2^. In a larger retrospective Mayo Clinic study where 180 patients underwent RYGB surgery, similar results were obtained. After a 3-year follow up, BMI reduction was observed from an initial value of 49 kg/m^2^ to 33 kg/m^2^ [23].

In terms of weight loss, we reported an average weight loss of 31.7 kg in men (median: 28.0 kg) and 29.3 kg in women (median: 27.0 kg) 6 months after surgery. Similar results were observed in the Schauer study, where the average weight loss obtained one year after RYGB was 29.4 kg, while for patients who had sleeve gastrectomy (SG) surgery, it was 25.1 kg, compared to only 5.4 kg in the untreated population [24]. Likewise, in our study, the persistence of weight reduction at the 5 years’ time point following laparoscopic intervention was monitored.

Importantly, bariatric surgery in our study group was found to lead to significant and sustained weight loss with beneficial effects on certain associated co-morbidities, as well to a reduction in certain risk factors for cardiovascular disease. After surgery, we found a significant decrease in the number of patients with Type 2 diabetes and insulin resistance. In the initial cohort with 36% of patients who had diabetes at baseline, T2DM persisted only in 14% of them 6 months after surgery [12,13].

Blanc et al. reported [25] complete remission of diabetes mellitus in 38% of patients one year following bariatric surgery. RYGB surgery is thought to have a direct anti-diabetic effect. Potential mechanisms underlying the anti-diabetic effect of RYGB include enhanced stimulation of intestinal hormones from the lower bowel (e.g., glucagon-like-peptide 1 hormone (GLP1)), altered bowel physiology with exclusion of nutrients from the upper bowel, compromised and decreased ghrelin secretion, modulation of insulin sensitivity, and other changes that are not fully characterized [26]. Elevated GLP-1 levels clearly enhance insulin secretion but may also lead to beta cell mass expansion, which some researchers believe leads to hyperinsulinemic hypoglycemia after RYGB [27]. Our results indicate a decrease of T2DM prevalence, as well as a reduction in the number of patients using antidiabetic drugs after bariatric surgery. We are still treating insulin resistance in only one patient after 5 years, with metformin, although her fasting glucose values are normal. She was prescribed that drug by her primary care physician due to polycystic ovary syndrome according to the homeostasis model assessment (HOMA). Medications that reduce insulin resistance include biguanides and thiazolidinediones, which have insulin-sensitizing and antihyperglycemic effects [28].

Pories and coauthors [29] reported long-term outcomes in 608 patients with extreme obesity who underwent RYGB surgery, with a follow-up rate of 93% of patients over 14 years. In addition to achieving significant, long-lasting weight loss, these individuals also experienced significant T2DM remission, such that of 146 people who had diabetes at baseline, 83% achieved normoglycemia, meaning no antidiabetic drug therapy. A meta-analysis of 136 studies of bariatric surgery, including 22,094 people, confirmed remission of T2DM after RYGB [30] similar to the aforementioned findings.

In a study by Buchvald et al. [30], hypertension was reduced in 61.7% of patients after bariatric surgery. Blank [25] reported that after one year of follow-up following SG, a reduction of hypertension was observed in 33% of the subjects. Our study did not show a decrease in the number of hypertensive patients or a reduction in the use of antihypertensive drugs after bariatric surgery. Although the same number of patients used pharmacotherapy for hypertension pre- and post-operatively, in some patients who had bariatric surgery, the overall number of antihypertensive medications decreased [13].

Our patients significantly improved their level of physical activity after bariatric intervention. Many of them replaced a sedentary lifestyle with a more physically active one. From the initial 34.8% of the participants who were considered physically active before the intervention, physically active patients increased to almost 64% 6 months after surgery [13]. Physical activity was defined by having daily walks of a minimum one hour by self-report. Physical exercise pre- and post-operation should be recommended as a beneficial add-on therapy following bariatric surgery. A systematic review published in 2012 suggested that exercise after bariatric surgery increases weight loss by an average of about 3.5 kg [31]. Results from a number of studies show that regular physical activity is one of the most important predictors of continuous weight loss after surgery [32]. Bariatric surgery leads to significant weight loss, improves metabolic status, and is considered the most effective treatment for morbidly patients with obesity.

In Olbers’ study [33] of 78 participants with obesity, only 15% were initially hypertensive, with all patients recorded as normotensive 5 years after surgery. The percentage of patients with dyslipidemia decreased from 69% to 15% after 5 years from the start of the study, while no deaths were recorded. The results of the aforementioned SOS study [15] showed that the prevalence of T2DM decreased significantly 2 and 10 years after surgery in the surgical group compared with the control group, while the incidence of hypertension and hypercholesterolemia did not differ significantly between the groups during the same period. After 2 years from surgery, 72% of patients became normoglycemic compared to 21% in the control group. At the 10-year follow-up, T2DM remission was reported in 36% of surgical patients compared to 13% of patients in the control group [15].

## 5. Conclusions

In a 5-year study of 66 patients who underwent bariatric surgery for obesity in Belgrade, Serbia, we demonstrated a positive long-term impact of bariatric surgery as well as notable and sustainable weight loss and BMI decrease which were associated with reduction of co-morbidities and risk factors for cardiovascular diseases. In our study population, there was a significant decrease in T2DM, affecting initially 36% of the subjects and, 5 years following gastric bypass surgery, 14% of them. In addition, a significant increase in physical activity level was also noted in our population, with 72% of patients considered as being physically active 5 years after surgery. Also, the number of patients using antidiabetic drugs was significantly reduced. Of our initial cohort of 66 patients, only 3% of the participants reported no weight loss after surgery. 

Considering the long-term quality of life of patients with obesity, as well as health costs [34,35], these are very important findings. Nevertheless, more studies with a larger number of participants are needed for further analyses and assessments of variations that may occur in this population.

## 6. Limitations of Study

The number of patients included and followed up postoperatively was relatively small, but comparable to those of previously published studies [36,37]. Our study population was predominantly female, so future studies including more male participants are needed in order to better understand whether weight loss is maintained equally in men and women. The lack of a control group is a limitation. Nevertheless, the main focus of this study was to check the long-term effect of bariatric surgery, that is, whether initial post-operative results in our cohort of patients were maintained after 5 years of follow-up. The findings of this observational study should be confirmed by randomized controlled trials with a large sample size. 

## Figures and Tables

**Table:** 

One-year overall survival	100%
Three-year overall survival	100%
Five-year overall survival	96.97%

**Table 1:** Number of patients experiencing weight loss 5 years after bariatric surgery (5 years versus baseline and 5 years versus 6 months after surgery).

Weight Loss	Five Years Versus Six Months after Surgery		*p* Value ^1^
	No	Yes	
6 months (*n* = 64)	2	62	<0.001
5 years (*n* = 62)	24	38	

^1^ McNemar test.

**Table 2:** Patient outcomes before and after bariatric surgery according to co-morbidities and therapy.

Number (%)	Before Surgery	Five Years after Surgery	*p* Value ^1^
Co-morbidities:			
Physical activity: active	23 (34.8)	46 (71.9)	<0.001
Hypertension: yes	29 (43.9)	23 (35.9)	0.452
OSA: yes	2 (3.0)	-	0.490
T2DM: yes	24 (36.4)	9 (14.1)	0.005
Hypercholesterolemia: yes	6 (9.1)	7 (10.6)	1.000
Hypertriglyceridemia: yes	6 (9.1)	5 (7.8)	1.000
Smoking: yes	14 (21.2)	22 (33.3)	0.193
Therapy:			
Antihypertensive therapy: yes	26 (39.4)	21 (32.8)	0.681
Beta blockers: yes	16 (24.2)	10 (15.6)	0.313
Ca antagonist: yes	11 (16.7)	5 (7.8)	0.204
ACE inhibitors: yes	17 (25.8)	17 (26.6)	1.000
Antidiabetics: yes	24 (36.4)	10 (15.6)	0.013

^1^ Chi-square test; OSA, obstructive sleep apnea syndrome, T2DM, diabetes mellitus type II, ACE inhibitors, angiotensin-converting-enzyme inhibitors; Ca antagonist, Calcium channel blockers; in our study, hypercholesterolemia (3.6–5.1 mmol/L) was defined as total serum cholesterol above 5.1 mmol/L, normal triglycerides corresponded to 0.2–1.8 mmol/L, hypertriglyceridemia corresponded to levels of triglycerides above 1.8 mmol/L, and arterial hypertension was defined as blood pressure above 140 and/or 90 mmHg.
